# Rheological and Curing Behavior of Acrylate-Based Suspensions for the DLP 3D Printing of Complex Zirconia Parts

**DOI:** 10.3390/ma11122350

**Published:** 2018-11-22

**Authors:** Dmitrii A. Komissarenko, Petr S. Sokolov, Anastasiya D. Evstigneeva, Irina A. Shmeleva, Alexey E. Dosovitsky

**Affiliations:** 1 NRC “Kurchatov Institute”-IREA, Bogorodskiy val str. 3, Moscow 107076, Russia; sokolov.petr@gmail.com (P.S.S.); aevstigneeva@mail.ru (A.D.E.); iriana9@rambler.ru (I.A.S.); 2 “NeoChem” JSC, Profsoyuznaya str. 115-2-331, Moscow 117647, Russia; dossov@com2com.ru

**Keywords:** additive manufacturing, ceramic, digital light processing, stereolithography, zirconia

## Abstract

The present study demonstrates the possibility of fabricating zirconia parts with a complex shape and internal architecture using a low-cost stereolithography-based technique. One of the critical steps in ceramics stereolithography is the preparation of a photo-curable slurry with properties that fulfill specific requirements, such as having a low viscosity, high solids loading and appropriate curing characteristics. Slurries with different acrylic monomers and ceramic fillers were studied concerning their rheological and curing behavior. New formulations based on mono- and tri-functional acrylic monomers revealed the following excellent rheological properties: The viscosity of the mono-/tri-acrylate-based slurry with 75 wt.% of zirconia was 1.6 Pa·s at 30 s^−1^. Zirconia stabilized with 3 mol.% yttria was found to be more favorable than zirconia with 8 mol.% yttria for slurry preparation, because of its lower surface area and higher tapped density. It was shown that the cure depth of the suspensions was suitable for printing objects with a 50 µm layer thickness, good interlayers connection and surface finishing.

## 1. Introduction

Stereolithography is one of the most promising additive manufacturing techniques as it allows for the fabrication of ceramic parts with a complex shape and precisely controlled internal architecture [1,2,3,4,5,6]. The method is based on the layer-by-layer photopolymerization of a liquid resin filled with ceramic particles directly from the CAD model and, is known for its high accuracy and excellent surface finishing [5,6,7,8,9,10]. There are two types of stereolithography-based techniques that can be applied to fabricate 3D ceramic objects. In the laser configuration (SLA), photopolymerization occurs via selective ultraviolet (UV) light exposure onto a liquid monomer, whereas in digital light processing (DLP), a projector illuminates the complete area at once. After the creation of a 3D object, the polymeric matrix has to be removed from the green body by thermal treatment and then the binder-free object can be sintered at evaluated temperatures.

The typical composition of the resin used in SLA and DLP involves UV-curable monomer/oligomer, a photoinitiator, diluents, and different additives for the stabilization of ceramic particles. Ceramic suspensions used in stereolithography must fulfil specific requirements. The viscosity of the suspension should not exceed 3 Pa·s in order to achieve a good layer recoating and self-levelling [11,12]. The suspension must exhibit a sufficient cured depth at the operating conditions in order to provide adequate integration between the layers and to ensure the precision of 3D printing [13]. Additionally, in order to achieve a good densification of a ceramic body, the suspension should contain as much ceramic particles as possible [12]. However, when ceramic particles are added into organic media, they induce light scattering on the particles’ surface, which subsequently leads to a reduction of the cured depth. Moreover, the introduction of ceramic powder into a resin drastically increases the viscosity of the suspension.

Zirconium dioxide has higher density (6 g/cm^3^), and higher refractive index (n20/D ~ 2.2) compared with silica or alumina, which complicates the 3D printing process. The large difference between the refractive index of monomer and particles leads to a reduction in the penetration depth of the UV-beam. For instance, the cured depth of suspension loaded with zircon was found to be four times lower than that of the suspension with silica fillers at equal operating conditions and solid content [14]. Masciandaro et al. [15] successfully printed self-supported electrolytes of yttria-stabilized zirconia using highly viscous pasts and Ceramaker SLA printing machine. Water-based slurries containing acrylamide were used to print zirconia dental bridges [16]. It was shown that the impregnation of the green body by an additional amount of zirconia could increase the density of the sintered body by up to 98.5% from a theoretical one. Mitteramskogler and Gerald [17] investigated the effect of curing conditions on the properties of the zirconia parts printed using digital light processing. It was found that the light scattering within the ceramic filled slurry caused a certain amount of widening of the dimensions in the final geometry and that this overgrowth is sensitive regarding both the overall exposure area and exposure time. A curing strategy with a low energy dose at the beginning of the fabrication process was proposed so as to eliminate the cracks formation in the green part. These observations were made with highly loaded and viscous slurries. Homa and Schwentenwein have also used highly viscous pasts (56 Pa·s at 50 s^−1^) to obtain dense zirconia parts with a stereolithography-based Lithoz machine [18]. The relative density of zirconia was found to be 99.1% and the flexural strength was 715 MPa, according to a three-point bending test. He et al. [19] fabricated triangular zirconia cutting tools using a DLP projector and a complex organic components mixture. The scanning electron microscope (SEM) analysis indicated that the parts were composed of highly dense submicron-grade grains. Although, it was found that the obtained zirconia tools were somewhat less dense than the zirconia ceramics prepared via the conventional method.

Most of the mentioned works have demonstrated success in the 3D printing of high-performance zirconia ceramics with expensive machines operating the highly viscous pasts. The present work aims to explore the possibility of printing complex stabilized zirconia parts on the commercial low-cost 3D printer.

## 2. Materials and Methods

### 2.1. Starting Materials

The ceramic material used in this work is zirconium dioxide stabilized with 3 and 8 mol.% of Y_2_O_3_, named 3YSZ and 8YSZ, respectively. High purity stabilized zirconia powders with a refractive index of 2.2 were synthesized using the co-precipitation method from concentrated aqueous solutions of zirconium oxychloride, yttrium nitrate, and ammonia. After the drying of the zirconium hydroxide, the precursor was ball milled and calcined at 1000 °C in the furnace for several hours. Before this, the slurries making zirconia powders were kept at 100 °C so as to prevent moisture absorbing on the particles’ surface.

The UV curable monomers used in this study were of technical grade—1,6-hexanediol diacrylate (HDDA), 1,1,1-trimethylolpropane acrylate (TMPTA), isobornyl acrylate (IBOA), 2-hydroxyethyl acrylate (HEA), 2-hydroxyethyl methacrylate (HEMA), 2-phenoxiethyl acrylate (PHEA), and isodecyl acrylate (IDA). Acrylate monomers usually contain 100–300 ppm of 4-methoxyphenol as an inhibitor of the radical polymerization. The detailed properties of the acrylic monomers at ambient conditions are presented in Table 1. The supplier was Abcr Gmbh (Karlsruhe, Germany), except for IDA and PHEA, provided by Sigma Aldrich (St. Louis, MO, USA). To initiate the polymerization reaction, ethyl (2,4,6-trimethylbenzoyl) phenylphosphinate was used. In order to achieve a good wetting and high dispersion of the ceramic particles in the organic media, the following surfactants were evaluated: BYK w969 and BYK w996, provided by BYK Additives & Instruments (Wesel, Germany); Triton X-45 and Triton X-114, provided by Sigma Aldrich (St. Louis, MO, USA). Triton X is a nonionic surfactant that has a hydrophilic polyethylene oxide chain and an aromatic hydrocarbon hydrophobic group. Whereas BYK w969 is a mixture of 2-phenoxyethanol and alkanolammonium salt of an acidic polyester and BYK w996 is a solution of a copolymer with acidic groups.

### 2.2. Suspension Preparation

The UV-curable slurry was prepared as follows: the mixture of acrylic monomer, surfactant and photoinitiator of 1 wt.% with respect to the monomer weight were blended in a polypropylene bottle; then zirconia powder was incrementally added into the organic media until the desired solid loading level has been reached. A homogeneous mixture was prepared using a SpeedMixer DAC 400.2 VAC-P (Hauschild, Germany). The SpeedMixer is based on the double rotation of the mixing cup (“dual asymmetric centrifuge”). The mixing parameters were 2 min at 800 rpm and 1 min at 2000 rpm followed by vacuum pumping at 10 mbar. In order to deaggregate, ceramic powder zirconia balls with a diameter of 1.5 mm were also placed into the polypropylene bottle.

### 2.3. Characterizations

The specific surface area of the as-prepared powders was determined using a TriStar 3000 analyzer (Micromeritics, Norcross, GA, USA) at a temperature of −195.8 °C, using a Brunauer–Emmett–Teller (BET) method. The powder particles’ morphology was studied using a JSM 7100 F scanning electron microscope (SEM) (Jeol, Tokyo, Japan). The particles size distribution was measured by laser diffraction using a MasterSizer 2000 (Malvern instrument, Worcestershire, UK). The powders’ tapped density (ρ_tap_) was determined according to the ISO 3953. The maximal error in the ρ_tap_ calculations did not exceed 0.04 g/cm^3^.

The rheological behavior of the suspensions was studied using rheometer Physica MCR 52 (Anton Paar, Graz, Austria). A plate-and-plate geometry with a measuring disk with a diameter of 25 mm and a gap of 0.200 mm was used. The viscosity was measured as a function of the shear rate in a region of 10–200 s^−1^ under isothermal conditions (20.0 °C). The viscosity versus shear rate curves were fitted (dashed lines) with an expression for a power law fluid. The flow parameters were calculated with the least square method.

The cure depth was determined via the polymerization of a thin layer of the suspension using a digital light projector of the Ember Autodesk printer, under room conditions (20–25 °C). After polymerization, the cured circle films were washed from the residual suspension in an inert solvent. The thickness of the polymerized films was measured using a micrometer. The uncertainty of each thickness measurement was estimated as 0.05 mm. Four independent thickness measurements were performed for each exposure time, and then the obtained data were averaged. A series of filaments cured at different exposure time (4.0–16.0 s) were used to build a curing profile.

The thermogravimetric analysis (TGA) and differential scanning calorimetry (DSC) of the cured suspensions were carried out on a simultaneous analyzer SDT Q600 (TA Instruments, New Castle, DE, USA), at a constant heating rate of 2 °C/min from an ambient temperature up to 600 °C, under dynamic air atmosphere. Images of the green and sintered bodies were taken on the SU1510 scanning electron microscope (Hitachi, Japan). The hardness of the ceramic samples was estimated using the Vickers method.

### 2.4. Fabrication of 3D Parts

A commercially available and low-cost 3D printer Ember (Autodesk, SAN Rafael, CA, USA) was used in the present work to fabricate ceramic objects with a complex shape. The Ember uses a 3D printing method in vat photopolymerization known as digital light processing. The main difference between this and standard laser stereolithography is the digital light projector that is used as a UV light source. The projector irradiates light below the vat through an optical window. The optical window in the bottom of the vat has a layer of polydimethylsiloxane (Elastosil RT 601) with a fluorinated ethylene propylene (FEP) film cover. The projector has a wavelength of 405 nm with a total optical power of 5 W. The Ember’s UV light source was monitored using a special UV light meter, the model 222 (G&R Labs, Santa Clara, CA, USA). Ember has a resolution of 50 μm [20] in the plane parallel to the printing surface (XY resolution). 

The 3D models have been designed using the Fusion 360 v2.0.4860 (Autodesk) CAD software. Print Studio v1.6.5 (Autodesk, SAN Rafael, CA, USA) was the software used for slicing and creating the print files. After printing, the green body was rinsed with an initial monomer in an ultrasonic bath and dried at room conditions for about three days. The debinding of the green body was performed with a heating rate of 1 °C/min up to 800 °C in air. Finally, sintering was conducted at 1600 °C for 2 h in air.

## 3. Results and Discussion

### 3.1. Powder Characteristics

**Figure 1:**
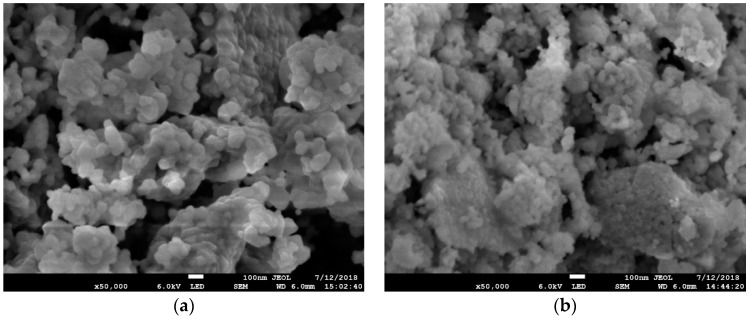
SEM micrographs of synthesized powders: (**a**) zirconium dioxide stabilized with 3 mol.% of Y_2_O_3_ (3YSZ); (**b**) zirconium dioxide stabilized with 8 mol.% of Y_2_O_3_ (8YSZ).

The characteristics of the ceramic fillers, such as the morphology, specific surface area, or particles size distribution, can directly influence on the properties of the suspension, and, subsequently, the quality of the green body and the final sintered part. Some essential characteristics of the synthesized powders are presented in Figure 1 and Table 2. It can be seen that zirconia stabilized with 8 mol.% of yttria has a slightly higher specific surface area compared with the tetragonal powder. On the contrary, the tapped density of 8YSZ and 3YSZ are 1.5 g/cm^3^ and 1.8 g/cm^3^, respectively. These values were obtained by mechanically tapping a certain amount of powder in a cylinder until no volume change was observed. The tapped density may be an important characteristic indicating how dense the particles could be compact in a slurry. The same trend in the decrease of the tapped density of zirconia with an increase of yttria content was also observed in the previous study [21].

According to the SEM, the tetragonal zirconia powders consist of round shape particles with a size of 100 nm. The primary particles are assembled into aggregates with the size close to 1 µm. Despite the equal synthesis conditions and the thermal treatment procedure, the particles of the 8YSZ powder have an irregular shape and a size of 50–70 nm. The laser diffraction analysis revealed that both 3YSZ and 8YSZ have a similar distribution of particles with a medium size (d_50_) of 1 µm.

### 3.2. Rheological Behavior of YSZ Suspensions

**Figure 2:**
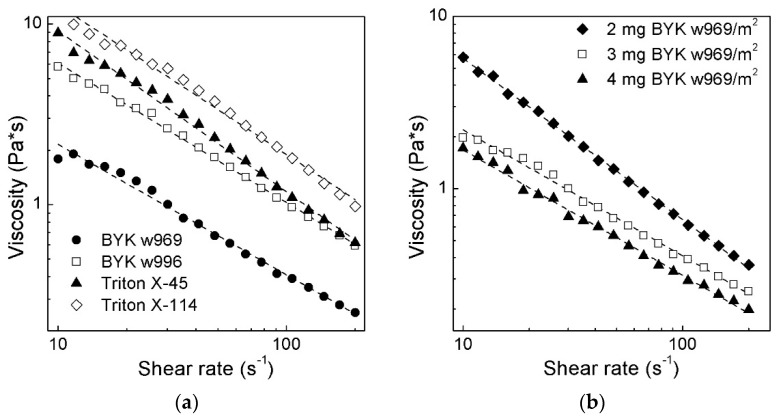
Viscosity of HDDA suspensions as a function of shear rate with 20 vol.% of 8YSZ: (**a**) influence of different type of surfactants; (**b**) influence of BYK w969 concentration at 20 °C.

The preparation of the slurry with appropriate characteristics is one of the central steps in ceramic stereolithography. Ceramic particles such as alumina, silica, and zirconia typically have hydroxyl groups on their surface, which are responsible for their hydrophilic nature [22,23,24,25]. Thus, to form a stable suspension with hydrophobic monomers, the modification of ceramic particles is essential. In the present study, four commercial surfactants (BYK w996, BYK w969, Triton X-45, and Triton X-114) were estimated as the wetting and dispersing agents (Figure 2a,b). Figure 2a shows the viscosity versus shear rate curves of the HDDA suspensions filled with 20% of 8YSZ powders and different types of surfactants. The concentration of the dispersant is presented as the amount of dispersant in mg, related to the specific surface area unit of the zirconia powder. 1,6-hexanediol diacrylate was chosen as the conventional monomer to be used in the stereolithography of many ceramic materials such as alumina [24], yttrium aluminum garnet [6], or calcium phosphate [5,26].

As it was observed, the type of surfactant can drastically change the viscosity of a suspension. In all cases, the suspensions revealed a shear thinning behavior. The calculated power law parameters (see Table S1) also indicated that the suspensions have a shear thinning behavior within the shear rate region of 10–200 s^−1^. Both Triton X-45 and Triton X-114 showed insufficient viscosity for the 20 vol.% of the solid loading. In both cases, the viscosity exceeded 3 Pa·s at a shear rate of 10 s^−1^. BYK w996 indicated the viscosity of 6 Pa·s at a shear rate of 10 s^−1^. In contrast to these surfactants, BYK w969 showed excellent rheological properties suitable for the ceramic stereolithography—at a shear rate of 10 s^−1^ the viscosity was less than 2 Pa·s, and dropped down to 0.3 Pa·s at a shear rate of 100 s^−1^.

Figure 2b shows the influence of the amount of BYK w969 on the rheology of the 1,6-hexanediol diacrylate filled with 20% 8YSZ. For BYK w969, the increase of the ceramic powder from 2 mg/m^2^ to 3 mg/m^2^ leads to a remarkable decrease in viscosity. A further increase in the amount of BYK w969 in the suspension did not lead to a significant decrease in the viscosity. Moreover, some variation behavior was observed between 20 and 30 s^−1^ for the curve with 4 mg BYK w969. Similar phenomena were also observed for the HDDA-based slurries with alumina fillers [24]. The observation is common for monodisperse particle suspensions, where, after a critical shear rate, the formation of jamming clusters occurs by hydrodynamic lubrication forces. At these shear rates, shearing force dominates over the Brownian motion, which pushes the particles into close proximity, where short-range hydrodynamic forces prevail. Therefore, higher forces are required to overcome this hydro-dynamic force, and thereby the suspension exhibits some variation from the shear thinning behavior. Basically, carboxylic acids considered the classical surfactants used for stabilization of ceramic particles in organic media [22,23,24]. However, using carboxylic acid as surfactants means it is sometimes complicated to achieve a very low viscosity at low values of shear rates.

BYK w969 was found to be an excellent surfactant suitable for slurry preparation with YSZ fillers. Moreover, the surfactant stabilized the particles against sedimentation needed for stereolithography process for a while. The unsatisfying behavior of the Triton X surfactants and BYK w996 is required for further investigation.

**Figure 3:**
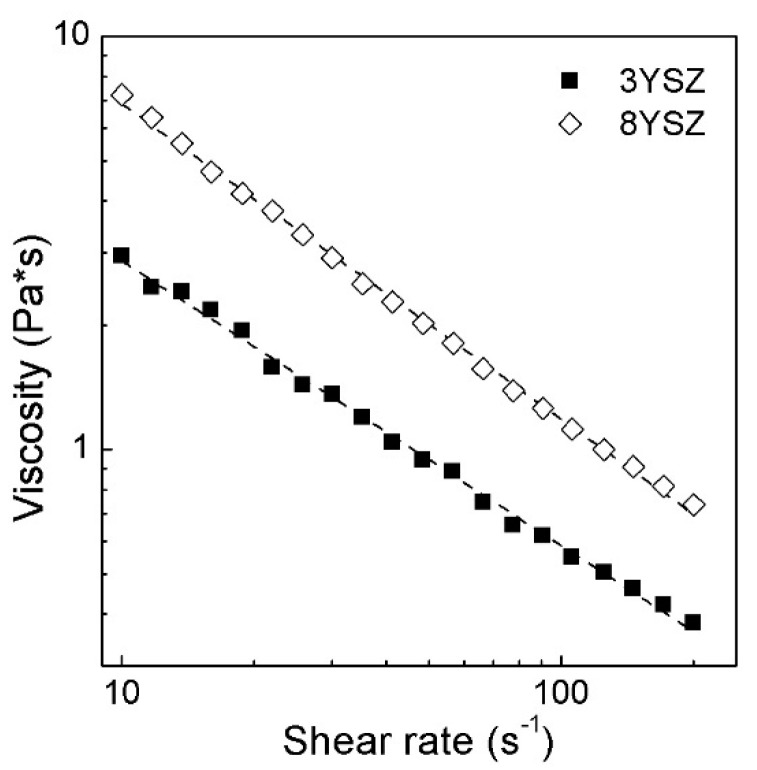
Viscosity of HDDA suspensions as a function of shear rate with two types of zirconia fillers (φ = 28 vol.%; surfactant BYK w969) at 20 °C.

To evaluate the influence of the yttria content in the zirconia fillers on the rheological behavior of suspensions, zirconia stabilized with 3 and 8 mol.% of Y_2_O_3_ were chosen for further experiments. Figure 3 shows the viscosity curves of the HDDA suspensions with 28 vol.% zirconia stabilized with 3 and 8 mol.% of Y_2_O_3_. For both of the suspensions, of 3YSZ and 8YSZ, the viscosity decreases with the increasing of shear rate which indicates a shear thinning behavior of dispersions appropriate for stereolithography-based processes. For the HDDA suspension with 8YSZ, evolution of the shear rate from 10 s^−1^ to 100 s^−1^ leads to a decrease of viscosity from 7.2 Pa·s to 0.7 Pa·s. At the same time, the viscosity of the suspension with tetragonal zirconia is almost three times lower than that of the 8YSZ suspension.

The difference in the viscosity of the 8YSZ and 3YSZ suspensions might be explained by the difference in the tapped density of the powders—as it was mentioned before, the tapped density of zirconia with 3YSZ is 1.8 g/cm^3^, while the density of 8YSZ is 1.5 g/cm^3^. Moreover, the specific surface area is decreasing with the decrease of the yttria content. The lower the surface area, the lower the amount of a surfactant needed for particles’ stabilization in a slurry.

The above mentioned experiments performed with 1,6-hexanediol diacrylate and BYK w969, showed a shear thinning behavior suitable for ceramic stereolithography. However, the introduction of zirconia particles with a solid loading higher than 28 vol.% led to a significant increase in the viscosity at low values of shear rate.

In order to increase the loading of zirconia in the organic media an attempt to replace conventional HDDA monomer to another system with lower viscosity has been done. A few acrylic monomers (HEA, HEMA, PHEA, IBOA and IDA) with monofunctional groups were chosen for this purpose. In stereolithography monofunctional monomers are generally used as reactive diluents because of their low viscosity. On the other hand, they can be potentially used as the main components for suspension preparation. For example, 2-hydroxyethyl acrylate with the addition of a small amount of PEGDA as a crosslinker was used to prepare highly concentrated nanosilica dispersion [25]. A transparent and dense silica part has been created after curing the suspension in a special chamber.

**Figure 4:**
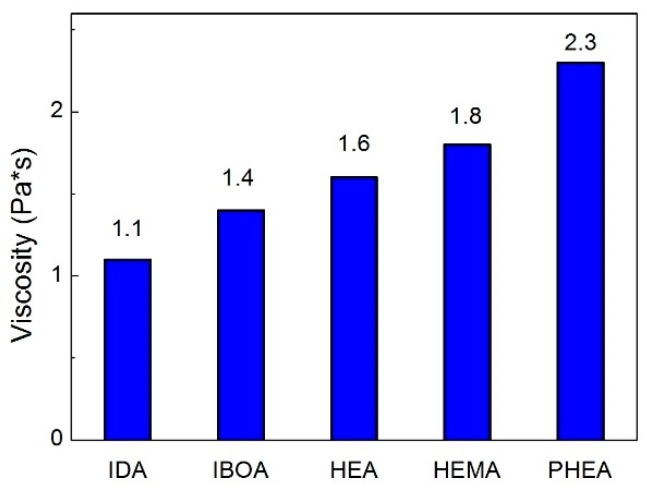
Viscosity of different acrylic monomers with 20 vol.% of 8YSZ at shear rate of 30 s^−1^ at 20 °C.

Figure 4 shows the viscosity of the monoacrylate suspensions with 20 vol.% of pure 8YSZ without the addition of any surfactant. Unexpectedly, HEA and HEMA did not reveal the lowest viscosity. It is known [20,23] that acrylic monomers with OH-terminated polar groups can interact with the particles’ surface, and can thus act as the surfactants. However, the lowest viscosity was found for IDA and IBOA.

The suspensions with IDA and IBOA indicated the lowest viscosity (1.1 and 1.4 Pa·s at shear rate of 30 s^−1^, respectively) and shear thinning behavior at either higher or lower shear rates among the studied monomers. The same trend was also observed with the additions of BYK w969. Note that pure monoacrylic monomers are quite difficult to cure. In this case, it is necessary to add some amount of crosslinking agent into a slurry. TMPTA was found to be a good component for facilitating the photocuring reaction [22].

Thus, IDA and IBOA with the addition of 15 wt.% of TMPTA as a crosslinking agent were chosen for the further rheological and curing experiments. This amount of TMPTA was found to be a compromise in terms of the viscosity and curing behavior.

**Figure 5:**
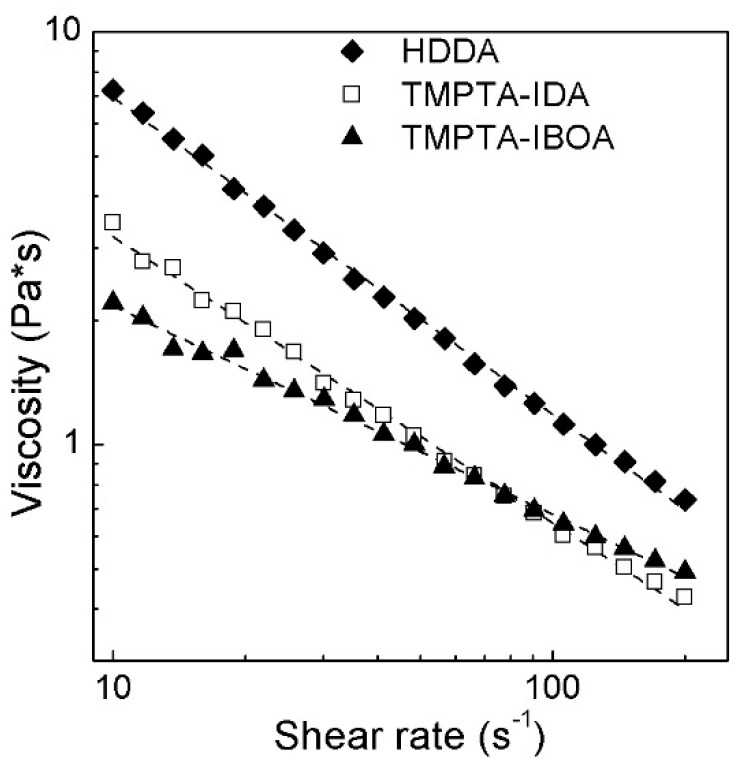
Viscosity of different acrylic monomers and their mixtures as a function of shear rate with 28 vol.% of 8YSZ at 20 °C.

As can be seen from Figure 5, the use of IDA and IBOA suspensions with addition of TMPTA can remarkably reduce the viscosity of the system. At the solid loading of 28 vol.% of 8YSZ, the viscosity of the suspension with IBOA-TMPTA media at 10 s^-1^ was three times lower (2.2 Pa·s) than that of the slurry with HDDA (7.1 Pa·s). The viscosity of IDA-TMPTA was the lowest for the highest values of the shear rates (0.1 Pa·s at 200 s^−1^). Hence, these slurries revealed an appropriate rheological behavior. To estimate the possibility of using the IBOA-TMPTA and IDA-TMPTA monomers in DLP 3D printing, further curing experiments have to be carried out.

### 3.3. Curing Behavior YSZ Suspensions with Different Monomers

The key parameter in terms of the reactivity of the UV curable suspension is the cure depth (D_p_), which is responsible for the layers’ connection. The polymerization depth (C_p_) is expressed by the Jacob Equation (Beer-Lamber law), as follows:

C_p_ = D_p_ln(E/E_c_)

where D_p_ is the depth of penetration, E is the exposure energy and E_c_ is the critical (or minimal) exposure energy needed to provide polymerization of the monomer. The introduction of ceramic particles into a monomer induces light scattering and limits the penetration depth of UV light.

**Figure 6:**
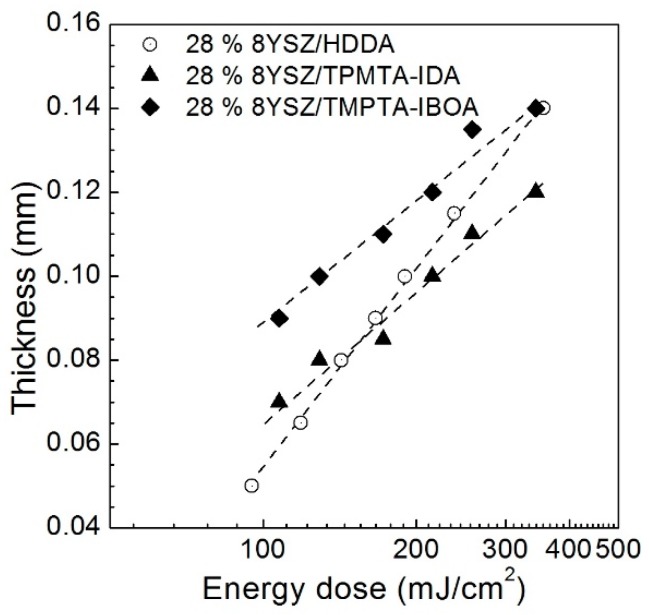
Layer thickness of the suspensions as a function energy dose applied.

Three suspensions with a 1 wt.% of photoinitiator, with respect to the HDDA, IDA-TMPTA, and IBOA-TMPTA monomers, were cured at a UV power of 21 mW/cm^2^ for various times. The solid loading was 28 vol.% of 8YSZ in all of the cases. Figure 6 shows the layer thickness of the cured films as a function of the energy dose. For the IBOA-based suspension the layer thickness grew up from 90 µm to 140 µm, with an increase of energy from 108 mJ/cm^2^ to 344 mJ/cm^2^, respectively. The found values suggest that the exposure time of 5.0 s which was equal to approximately 100 mJ/cm^2^ was enough to print 3D objects with a layer thickness of 50 µm. This thickness was proposed to be an optimum in terms of the appropriate resolution and time consumed to print the whole object. The values of the critical energies and the penetration depth of the suspensions calculated from the slopes of the curves are presented in Table 3.

The present values of the layer thickness are related to the suspensions filled with 28 vol.% of 8YSZ powder. It is known that the introduction of a solid into a pure resin remarkably decreases the cure depth of the suspension. However, it was found that the increase of zirconia content from 28 vol.% to 33 vol.% in the IBOA-TMPTA suspension did not lead to a significant decrease of penetration depth with 1% of photoinitiator. The difference in the polymerization kinetic of three slurries might be explained by the difference in the reactivity of the HDDA, IBOA, IDA, and TMPTA components. The acrylic monomers undergo radical polymerization after UV exposure, with the formation of three radicals and their migration through organic media. The curing profile had a similar curve slope and layer thickness within a margin error (5 µm). It should be also noted that the comparison of 8YSZ and 3YSZ powders in terms of the suspension reactivity did not reveal any contribution of yttria content. Considering the fact that the 3YSZ suspensions showed better rheological characteristics with an equal solid content, the zirconia stabilized with 3 mol.% of yttria was chosen for the DLP 3D printing.

### 3.4. Thermal Debinding

**Figure 7:**
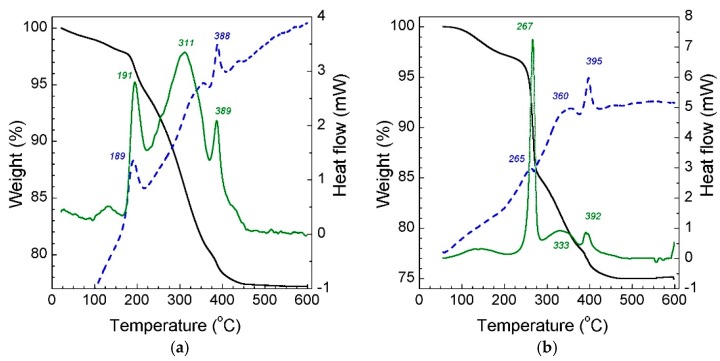
TGA (black solid curve), DTG (green solid curve) and DSC (blue dash curve) curves of the cured suspensions with 33 vol.% of 3YSZ: (**a**) IDA + TMPTA; (**b**) IBOA + TMPTA.

Thermal decomposition is one of the crucial steps in stereolithography-based 3D printing. The choice of an appropriate thermal debinding regime can significantly eliminate the formation of defects in the binder-free and sintered bodies. In order to build a thermal decomposition profile, small cured disks of suspensions filled with 33 vol.% of 3YSZ were taken for TGA-DSC analysis. The thermal decomposition curves of the slurries cured at the equal exposure time are shown in Figure 7.

For the IDA-TMPTA cured disks, the main weight loss between 190 °C and 450 °C was observed. The minor loss of 2 wt.% below 190 °C might be related to the evaporation of the uncured liquid monomers, surfactants or photoinitiator. Extensive peaks at 311 °C and 388 °C on the DTG curve are attributed to the decomposition of the copolymer of IDA and TMPTA. The decomposition of the IBOA-TPMTA polymer matrix starts to occur at a slightly higher temperature. A sharp weight loss was observed at 267 °C, indicating the rapid oxidation reaction of the matrix. The final weight loss of the two compositions is in a good agreement with solid loading. Few exothermic peaks can be observed on the DSC curves. The exothermic events of the process confirmed that the degradation involves oxidation reactions.

### 3.5. Formation of 3D Objects

**Figure 8:**
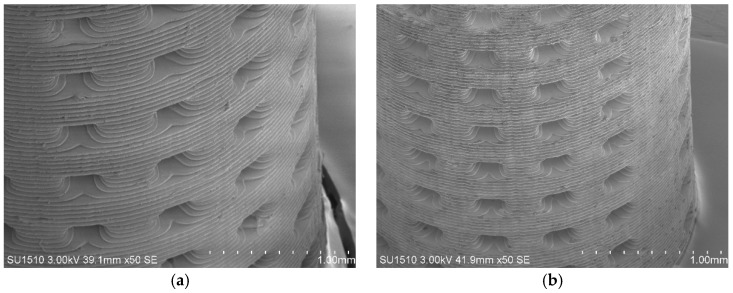
The fabricated parts derived from IDA-TMPTA suspension loaded with 33 vol.% of 3YSZ: (**a**) green body; (**b**) ceramic.

The fabrication of holey tubes with a layer thickness of 50 µm was carried out on the Ember desktop printer, with IDA-TMPTA and IBOA-TMPTA suspensions loaded with 33 vol.% of 3YSZ powder. The tube’s model had following characteristics: 6.00 mm high, 3.80 mm external diameter, and 0.30 mm wall thickness. The sizes of the green bodies after printing are 6.05(5) mm (high), 3.85(5) mm (dia.) and 0.35(1) mm (wall). The final sizes of ceramic tube are 4.25(5) mm (high), 2.80(5) mm (dia.) and 0.25(1) mm (wall). Thus, the average linear shrinkage was about 29 % (calculated anticipated value is 31%). One can conclude that the relative density of the 3YSZ ceramic is above 90%. The representative pictures of the green body and ceramic tubes are shown in Figure 8. Additional Figures S1–S5 are presented in the Supplementary Materials.

The properties of the green bodies derived from our slurries were satisfactory concerning the balance between their hardness and flexibility. The green tubes did not seem to undergo any visible delamination or other damage after ultrasonic treatment we used to wash out the residual monomer. They were suitable to print thin-walled ceramic tubes. The available wall thickness is limited not only by the composition of the slurry but also by the technical capabilities of the printer. It is complicated to manufacture a green body wall with a thickness of fewer than 200 microns. The upper limit of the wall thickness is also limited by the adhesion forces between the layers of polymer/FEP film, polymer/polymer and polymer/glass (material of the build head). At the same time, the solid support structures for the cylinders with a diameter of ~8 mm and ~4 mm in height were burned out accurately.

The printability of the 3D object with a light expose of 3 s and a layer thickness of 25 μm was also demonstrated (Figure S5). In this case, the time consumed to print the object increased from 32 to 54 min. It demonstrates that both of the chosen suspensions can be potentially be used to fabricate complex stabilized zirconia parts with satisfactory surface finishing, and without critical defects. At the same time, some delamination between the layers and small cracks on the IBOA-TMPTA-derived part can be observed. Almost no defects on the IDA-TMPTA green body were detected. The estimated Vickers hardness of the ceramic parts was 11 GPa (Figure S6).

## 4. Conclusions

The printability of yttria stabilized complex zirconia parts using new formulations based on mono- and triacrylates was investigated. Highly loaded zirconia slurries with a solid content of 75 wt.% were revealed to be suitable for stereolithography rheological and curing behavior. Isobornyl acrylate with the addition of TMPTA as a crosslinker showed the viscosity of 1.6 Pa·s at 30 s^−1^ with 33 vol.% of solid loading. Green zirconia bodies with an adequate surface finishing and without critical defects on the parts have been fabricated using a DLP printer.

## Figures and Tables

**Table 1:** Typical properties of the monomers selected for evaluation in this study at 20 °C.

Monomer	Molar Mass (g/mol)	Density (g/cm^3^)	Viscosity (mPa·s)	Refractive Index (n20/D)
HDDA	226.3	1.01	9	1.456
TMPTA	296.4	1.11	130	1.474
IBOA	208.3	0.99	2	1.475
HEA	116.1	1.011	8	1.445
HEMA	130.1	1.073	11	1.453
PHEA	192.2	1.104	10	1.518
IDA	212.3	0.875	5	1.442

**Table 2:** Characteristics of the synthesized zirconia powders.

Filler	BET (m^2^/g)	d_50_ (µm)	Tapped Density (g/cm^3^)	Refractive Index (n20/D)
3YSZ	6.5	1	1.8	2.2
8YSZ	12	1	1.5	2.2

**Table 3:** Calculated critical energies (E_c_) and penetration depth (D_p_) of the evaluated photocurable monomers with 28 vol.% of 8YSZ.

Monomer	D_p_ (µm)	E_c_ (mJ/cm^2^)	R^2^
HDDA	68(2)	45(4)	0.998
IBOA-TMPTA	44(2)	14(3)	0.978
IDA-TMPTA	43(2)	21(3)	0.979

## Supplementary Materials

The following are available online at http://www.mdpi.com/1996-1944/11/12/2350/s1, Table S1: Calculated power law parameters (dashed curves) for acrylate-based suspensions, Figure S1: SEM micrographs of green body derived from IBOA-TMPTA suspension (20× and 100× magnification), Figure S2: SEM micrographs of green body derived from IBOA-TMPTA suspension (20× and 100× magnification), Figure S3: SEM micrographs of 3YSZ ceramic derived from IBOA-TMPTA suspension (20× and 100× magnification), Figure S4: SEM micrographs of 3YSZ ceramic derived from IDA-TMPTA suspensions (20× and 100× magnification), Figure S5: SEM micrographs of green body derived from IBOA-TMPTA suspension with 25 µm layer thickness and the fabricated 3YSZ ceramic, Figure S6: SEM micrograph of a Vickers indent of 3YSZ ceramic, Figure S7: Optical image of green and sintered bodies derived IDA-TMPTA suspensions.
