# Supplementary material for: Rheological and Curing Behavior of Acrylate-Based Suspensions for the DLP 3D Printing of Complex Zirconia Parts
Source: Materials (Basel). 2018 Nov 22;11(12):2350. doi: 10.3390/ma11122350 (PMC6316993; doi:10.3390/ma11122350)
Supplement: Supplementary file 1 [file materials-11-02350-s001.pdf]

materials

Article

Rheological and Curing Behavior of Acrylate-Based Suspensions for the DLP 3D Printing of Complex Zirconia Parts

Dmitrii A. Komissarenko [1,*], Petr S. Sokolov [1], Anastasiya D. Evstigneeva [1], Irina A. Shmeleva [1] and Alexey E. Dosovitsky [2]

1 NRC "Kurchatov Institute"-IREA, Bogorodskiy val str. 3; Moscow, 107076, Russia; sokolov.petr@gmail.com (P.S.S.); aevstigneeva@mail.ru (A.D.E.); iriana9@rambler.ru (I.A.S.)
2 "NeoChem" JSC, Profsoyuznaya str. 115-2-331, Moscow, 117647, Russia; dossov@com2com.ru
* Correspondence: komissarenko.d@gmail.com; Tel.: +7-495-963-73-50

Table S1. Calculated power law parameters (dashed curves) for acrylate-based suspensions with YSZ powders and surfactants in a shear rate region of 10–200 s^{-1}.

Suspension	$Log_{10} K$	n-1	R^2
20% 8YSZ/HDDA/BYK w969—Figure 2a	1.05(3)	−0.72(2)	0.990
20% 8YSZ/HDDA/BYK w996—Figure 2a	1.56(1)	−0.77(1)	0.998
20% 8YSZ/HDDA/Triton X-45—Figure 2a	1.85(3)	−0.89(2)	0.994
20% 8YSZ/HDDA/Triton X-114—Figure 2a	1.93(3)	−0.93(2)	0.992
20% 8YSZ/HDDA/2 mg BYK w969—Figure 2b	1.69(1)	−0.94(1)	0.999
20% 8YSZ/HDDA/3 mg BYK w969—Figure 2b	1.07(2)	−0.73(1)	0.994
20% 8YSZ/HDDA/4 mg BYK w969—Figure 2b	0.95(2)	−0.73(2)	0.995
28% 8YSZ/HDDA/3 mg BYK w969—Figure 3	1.60(1)	−0.76(1)	0.998
28% 3YSZ/HDDA/3 mg BYK w969—Figure 3	1.15(2)	−0.69(1)	0.996
28% 8YSZ/IBOA-TMPTA/3 mg BYK w969—Figure 5	0.84(1)	−0.51(1)	0.995
28% 8YSZ/IDA-TMPTA/3 mg BYK w969—Figure 5	1.20(1)	−0.69(1)	0.996

The power law expression: $\eta = K\gamma^{n-1}$, where η is viscosity, γ is shear rate, K is a consistency index, n is power law index.

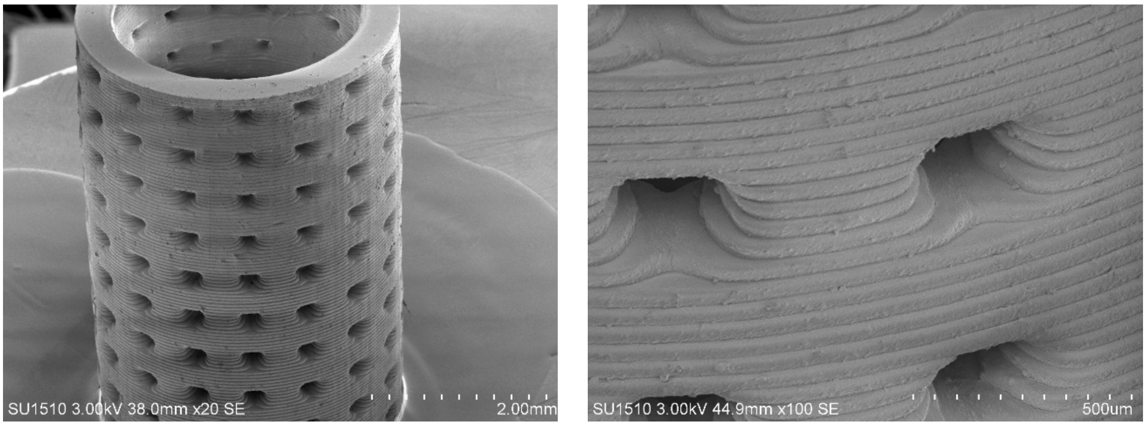

(a) **(b)**

Figure S1. The fabricated green bodies derived from IBOA-TMPTA suspensions with 33 vol.% 3YSZ (50 μm layer thickness)—(**a**) ×20 and (**b**) ×100 magnification.

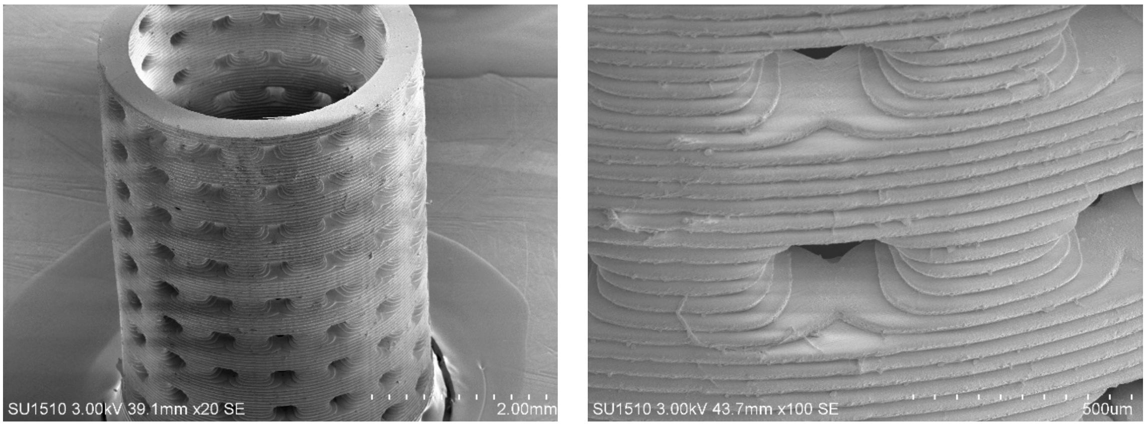

(a) **(b)**

Figure S2. The fabricated green bodies derived from IDA-TMPTA suspensions with 33 vol.% 3YSZ (50 μm layer thickness)—(**a**) ×20 and (**b**) ×100 magnification.

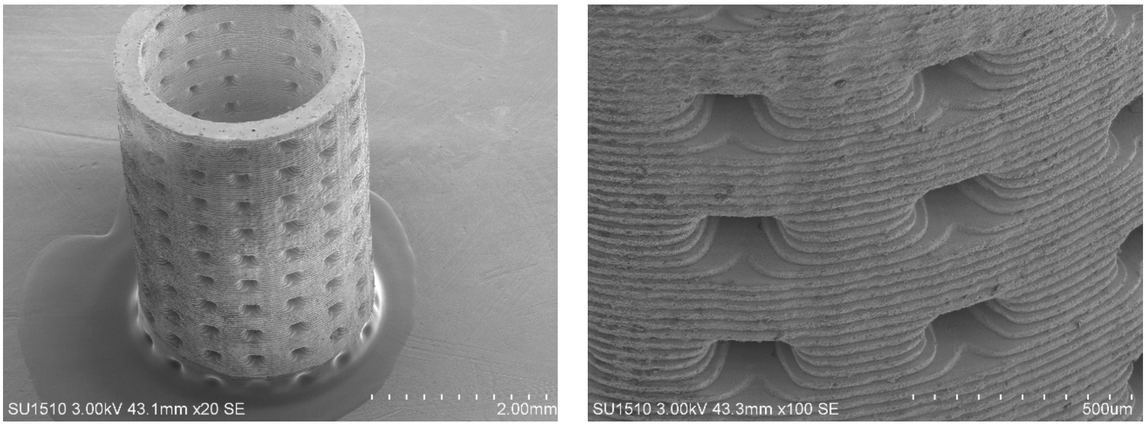

(a) **(b)**

Figure S3. The fabricated 3YSZ ceramic derived from IBOA-TMPTA suspensions (**a**) ×20 and (**b**) ×100 magnification.

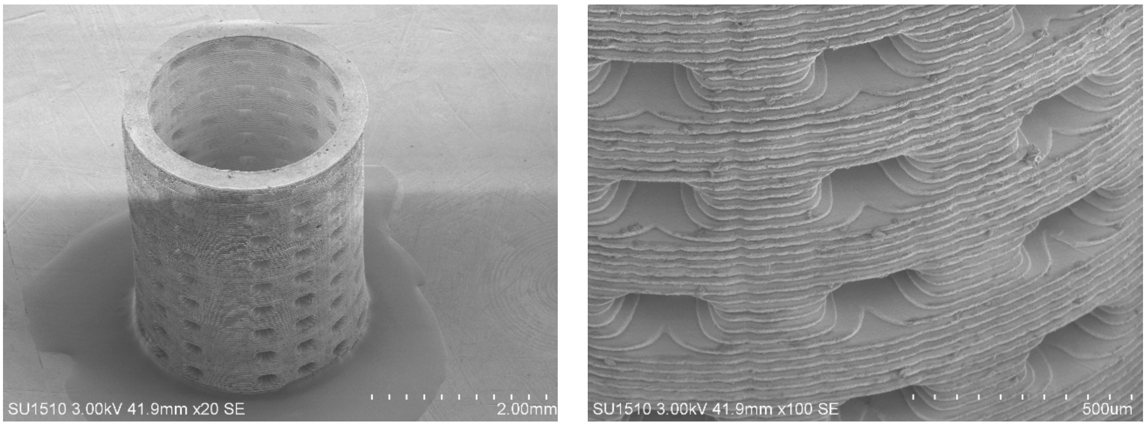

(a) **(b)**

Figure S4. The fabricated 3YSZ ceramic derived from IDA-TMPTA suspensions (**a**) ×20 and (**b**) ×100 magnification.

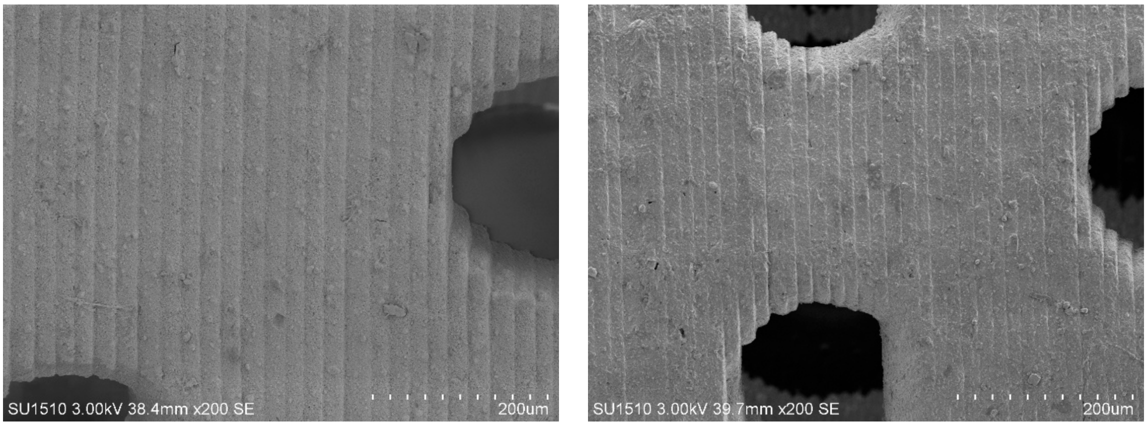

(a) **(b)**

Figure S5. (**a**) The fabricated green bodies derived from IBOA-TMPTA suspensions with 33 vol.% 3YSZ (25 μm layer thickness); (**b**) The fabricated 3YSZ ceramic.

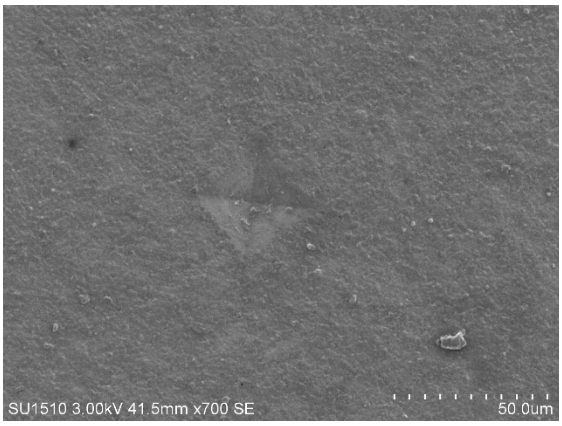

Figure S6. Representative SEM micrograph of a Vickers indent (1 kgf load) of 3YSZ ceramic.

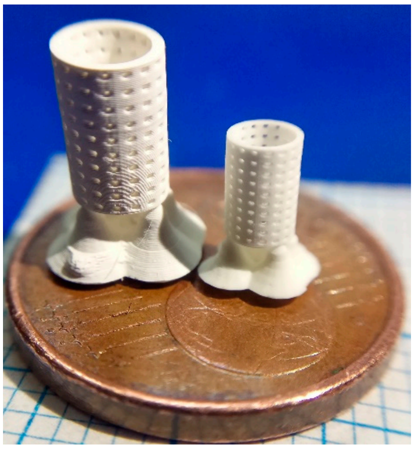

Figure S7. Optical image of green and sintered body derived IDA-TMPTA suspensions.

© 2018 by the authors. Licensee MDPI, Basel, Switzerland. This article is an open access article distributed under the terms and conditions of the Creative Commons Attribution (CC BY) license (http://creativecommons.org/licenses/by/4.0/).
